# Validity and Reliability of the Korean Version of the Work–Family Behavioral Role Conflict Scale (WFBRC-S)

**DOI:** 10.3390/ijerph17249273

**Published:** 2020-12-11

**Authors:** Mihyeon Seong, Ji Hyeun Song, Ji Sun Ha, Gi Jung Jung, Sohyune Sok

**Affiliations:** 1 Department of Nursing, Chang Shin University, Changwon-si 51352, Korea; mihyeon0624@cs.ac.kr; 2 Department of Nursing, Cheju-Halla University, Jeju-si 63092, Korea; kasili0809@chu.ac.kr; 3 Department of Nursing, Graduate School, Kyung Hee University, Seoul 02447, Korea; hajs@khu.ac.kr; 4 College of Nursing, Shin Han University, Uijeongbu-si 11644, Korea; gijung1004@naver.com; 5 College of Nursing Science, Kyung Hee University, Seoul 02447, Korea

**Keywords:** work, family, role conflict, validity, reliability

## Abstract

This study aimed to examine the validity and reliability of the Korean version of the Work–Family Behavioral Role Conflict Scale (WFBRC-S), which was originally developed to measure work–family behavioral role conflict in American adults with a wide variety of occupations such as nurses and chief executive officers. This study used a methodological research design. The study population consisted of 235 married men and women aged 20 years or older who were living in various cities, who had been employed for three years or more. The validity of the content, construct, convergent, discriminant, and criterion related, as well as the reliability of the WFBRC-S-K, was assessed. The WFBRC-S Korean version consists of 25 items. It was found that through the validity of the composition and standards of WFBRC-S-K, it was possible to measure the conflict by focusing on behavior so that a comprehensive evaluation of the role conflict between family and work, and work and family, can be performed. Five items in the WFBRC-S-K were excluded with a standardized factor loading of less than 0.50. We applied the modified index to improve the model fit to build a model, it supports a good fit and reliable score for the Korean version of the WFBRC-S model. Analysis of the fit of the revised model Nomed χ^2^ (CIMIN/df) value of less was: fit indices to 2.05 RMSEA = 0.07, RMR = 0.04, SRMR = 0.06, GFI = 0.85, IFI = 0.91, TLI = 0.90, CFI = 0.91. Criterion validity compared to the WLBOC-S showed significant correlation, and Cronbach’s alpha was 0.94. Factor loadings of the 25 questions ranged from 0.49 to 0.81. The study findings confirmed the applicability of this scale for measuring the work–family behavioral role conflict in Korean adults with a wide variety of occupations. The WFBRC-S-K can be applied on the measurement of work–family conflict in nursing and other industrial sites. These results provide a foundation for future studies on work–family behavioral role conflict in Korean adult.

## 1. Introduction

Work–family conflict will limit people’s vitality and wisdom when focusing on one role while not properly taking care of the other role, thereby causing psychological and behavioral conflicts [1]. Conflict between work and family is two-way because work interferes with the family and family interferes with work [2,3]. Several scholars and writers have raised concerns about the negative consequences of the work–family conflict [2,3]. In addition, current scholars point out that, when predicting work–family conflict, it is necessary to consider the cultural context and predict the conflict within families beyond the cultural dimension of collectivism and gender egalitarianism [2,3,4].

When South Korea entered the 2000s, the number of double-income families increased, and the country’s low birthrate continued to be cited as an important issue. These prompted major government ministries such as the Ministry of Health and Welfare and the Ministry of Gender Equality and Family to establish various institutional mechanisms in response, and academic research to be conducted [5]. In particular, as the number of double-income families increases, it has been reported that working hours of more than 50 h per week and increased use of smartphones for continuous connection to work are causing conflicts between work and family [6,7]. Traditionally, Korean families are strongly characterized by a male breadwinner model, where men work outside their homes to support their families while women take care of housework at home [8]. However, now that women’s participation in economic activities is becoming a necessity rather than an option, Korea is gradually shifting to a dual-earner model, and the boundary between the roles of men and women is gradually being blurred. As a result, a family culture is emerging differently from the past, such as men sharing housework with their spouse, and the balance between family life and work life is being re-established [9]. Double-income couples have difficulty in balancing their work–family life in the Korean society, thereby increasing the burden of childrearing and causing a serious social problem of low birth rate [10]. In particular, due to the residual influence of the traditional gender role norms that are slowly changing without reflecting the changes in women’s economic roles, double-income couples are having a lot of difficulty in reconciling their work life and their family life [11]. As a result, individuals cannot smoothly perform their roles between work and home, which causes stress and leads to mental health problems [12]. Thus, nursing discussion is required, but research is still lacking. Earlier, Europe had suggested, as a major goal, the solving of this work–life balance problem [9]. Thus, research on the conflict between work and family is very necessary nationwide.

According to previous studies [13,14,15,16], there are various concepts of the dimensions that cause conflict between work and family. Generally, however, the known concepts are time-based conflict (when the time required for one role makes it difficult to meet the requirements of another role), burden-based conflict (when the tension created in one domain makes it difficult to meet the requirements of another role), and behavior-based conflict (when the specific behaviors required by one role make it difficult to meet the requirements of another role). However, the existing tools for handling work–family conflict focus on the influence of individual perceptions on the family in terms of the dimensions of work–family conflict, and they need to be supplemented because they cannot distinguish between individual subjective behavior and behavioral role conflict [15].

Recently, in the field of nursing, role conflict has been defined as a state wherein people feel stressed in the process of performing a role related to their position in an organization, because the psychological cognitive states or behaviors of the person who performs the role conflict with those of the person in charge of the role [17]. According to Clark et al. [16], demands of work and family are often unacceptable, and with limited resources and the ever-increasing number of roles, more conflicts arise psychologically with the drive to succeed, which may increase the risk of having physical and/or mental health problems. However, despite this reality, in Korea, the degree of conflict is identified by analyzing data on surveys of time spent for family life of women and men in the early stage; variables such as the pressure of time use are used as substitute variables for work–family life conflict [18], or by asking the question “How successful do you feel about the work–family balance in work and family life?” [19]. The tools for dealing with work–family conflict that are used in Korea—including: the work–family conflict and family–work conflict scales developed by Netemeer, Boles, and McMurrian [20]; the work–family conflict scale for Korean women, validated by Yoo, Hong, Park, and Kim [21]; the work–family conflict scale by Carlson, Kacmar, and Williams [22]—are concentrated on women or were developed a long time ago. The existing tools perceived work to be more important than family, saw that what an individual could control in the area was extremely limited, and despite the fact that the family area was heavily affected by work, failed to distinguish the characteristics of work–family conflicts [23], thereby ignoring the family area values. They were single-direction measuring tools. In addition, there were no tools to examine conflicts in various areas, such as personal characteristics, work, family characteristics, role attitudes, etc., using a transfer theory that focuses on the interaction of the work–family conflict itself or tools that focuses on gender role attitude [24]. In addition, no tools have been developed to fit the current Korean situation and researched for reliability and validity in consideration of the three dimensions that cause conflict. Therefore, research on this subject is required.

To date, the measurement of work–family conflict has focused entirely on individual perception, thereby having the limits of distinguishing between the conflicts of an individual’s subjective role and behavioral role. Greenhaus et al. [25] reported that, regarding work–family conflict, the pressures are derived from each role from work and family domains, thus resulting in conflict. By separating the general state of work–family conflict from behavioral role conflict, the process of work and family can be more fully understood [26]. To this end, Clark et al. inductively developed work–family conflicts, including conflicts of behavioral roles, in order to complement the tool. According to Clark et al. [16], although existing tools adequately assess psychological role conflicts, conflicts when individuals use family time to perform work are not compatible with each other. Moreover, existing tools do not consider the conflict with specific behaviors from different roles that cause functional decline in a specific area. Therefore, Clark et al. [16] developed a tool for measuring the conflict by focusing on behavior so that the role conflict between family and work and between work and family could be comprehensively evaluated by supplementing the existing tools. That tool is the Work–Family Behavioral Role Conflict Scale (WFBRC-S). The WFBRC-S is meaningful in that it helps to fully understand work and family by separating the general state of work–family conflict from behavioral role conflict. This study uses Clark’s tool that reflects the dimension of conflicts of behavioral roles that may occur in the work–family area to see if the Korean version of the tool can be applied to Korean situations by verifying its validity.

In this study, the WFBRC-S was translated into Korean to fit the characteristics of Korean culture. Then, the validity and reliability of the Korean version was verified to determine if it is applicable to the Korean situation in terms of confirming and applying the work–family behavioral role conflict.

The purpose of this study was to verify the validity and reliability of the Korean version of Clark et al. [16]’s Work–Family Behavioral Role Conflict Scale (WFBRC-S). The aims of this study were to (1) verify the content validity of the Korean version of the WFBRC-S (hereinafter, WFBRC-S-K); (2) verify the construct validity, convergent validity, and discriminant validity of the WFBRC-S-K; (3) verify the criterion-related validity of the WFBRC-S-K; (4) verify the reliability of the WFBRC-S-K.

## 2. Materials and Methods

### 2.1. Study Design and Participants

This study was a methodological research that translates into Korean Clark et al. [16]’s WFBRC-S and verifies the validity and reliability of its Korean version. Double-income couples have difficulty in balancing work–family in the Korean society, increasing the burden of childrearing, and causing a serious social problem of low birth rate [10]. In particular, due to the remaining influence of the traditional gender role norms that are slowly changing without reflecting the changes in women’s economic roles, double-income couples are having a lot of difficulties in reconciling work and family [11]. Based on this, this study was conducted on 235 married men and women aged 20 years or older who were living in various cities, had been employed for three years or more, understood the purpose of this study, and agreed to voluntarily participate in this study. The sample size required for the validity test of the tool is four times the minimum number of items, so 200 or more subjects, comprehensively considering not only the number of variables to be measured but also the number of factors, their commonality, the factor loadings, etc. [27]. A total of 240 subjects, which is eight times the minimum number of items, were conveniently sampled. Questionnaires on the Korean version of the WFBRC-S, which consists of 30 items based on previous studies [27], were distributed to the subjects. A total of 238 accomplished questionnaires were collected (collection rate: 99.1%), from which the data of 235 subjects were used for the final analysis, excluding the data from the questionnaires of three subjects who responded insincerely.

### 2.2. Instruments

The Korean version of the work–family behavioral role conflict measurement tool (WFBRC-S) was developed and used by criticizing Clark et al. [16], who focused the measurement of general work–family and family–work conflicts only on individual perceptions, considering both directional conflicts of family–work and work–family, and translating and reverse translating the tools that were developed, including the conflicts of behavioral roles between work and family. Thus, it is necessary to verify the validity and reliability of the tool. This tool consists of two sub-dimensions that consider both directions, i.e., work–family conflict (Nos. 1–15) and family–work conflict (Nos. 16–30), with a total of 30 items. Each item is evaluated on a 5-point Likert scale, ranging from “Not at all” (1 point) to “Almost never” (2 points), “Average” (3 points), “Often” (4 points), and “Very often” (5 points). The higher the score is, the higher the level of conflict is. As to the reliability of the WFBRC-S, its Cronbach’s α was 0.90 at the time of its development and 0.93 for the entire length of this study. As for the sub-dimensions of the WFBRC-S, the Cronbach’s α of Work Interference with Family (WIF) and Family Interference with Work (FIW) was 0.91 and 0.90, respectively.

The work–life balance organizational culture measurement tool (WLBOC-S) is designed to measure the organizational culture to support the work–life balance of workers. This study used the tool that was developed by Park and Sohn [28] with its reliability and validity verified for the measurement. This tool consisted of a total of 22 items in five sub-dimensions: corporate will (5 items), superior consideration (5 items), peer communication (4 items), peer support (4 items), and ease of use of the system (4 items). Each item is evaluated on a 5-point Likert scale, ranging from “Not at all” (1 point) to “Almost never” (2 points), “Average” (3 points), “Often” (4 points), and “Very often” (5 points). The higher the score is, the more positive the organizational culture is. As to the overall reliability of the tool, its Cronbach’s α was 0.94 in Park and Sohn [28]’s study and 0.94 in this study. As for its sub-dimensions, the Cronbach’s α for corporate culture was 0.91; for superior consideration, 0.94; for communication, 0.91; for peer support, 0.93; for ease of use of the system, 0.81.

### 2.3. Procedures and Ethical Considerations

To confirm the reliability and validity of the WFBRC-S-K, this study conducted a questionnaire survey on office workers after obtaining approval from the Institutional Research Board of K University, South Korea (KHSIRB-19-244 (RA)). Data were collected from January 2020 to February 2020, and the subjects were asked to fill out the questionnaire anonymously through an Internet portal. The reason for using an Internet portal with a Google URL was that the current COVID-19 situation in 2020 limited the recruitment of subjects, and people avoided face-to-face contact, so the data were collected through an Internet portal that is often used by office workers. Before the data were collected, the study participants were informed about the purpose, procedure, and method of the study, and the questionnaire method, content, time required, and compliance with research ethics, specifically that the questionnaires would be sealed and the collected data would not be used for purposes other than the research, would be processed anonymously, and could be withdrawn at any time. Then, the researcher collected data only from those who agreed to voluntarily participate in the study. The questionnaires were self-reporting by study participants, and took about 15 min to complete.

### 2.4. Study Procedure

#### 2.4.1. Translation–Back Translation

In this study, the WFBRC-S was translated after obtaining approval from its developer, Clark et al. [16]. The original text was translated in four steps according to the translation–back translation procedure [29], i.e., according to the tool translation and application guidelines used to apply English tools in other cultures [29]. The validity of the translation procedure was secured by back-translating the first translation into the original language, comparing it with the original, and revising the differences. The translation process of the tool is as follows: (1) a person with more than 10 years of work experience and is fluent in two languages translated the original text into Korean; (2) the researcher and two nursing professors reviewed the translation by comparing it with the original to see if the translated version is appropriate for the Korean situation; (3) one professor at the nursing college with a doctoral degree in nursing and who is fluent in English and Korean, and one office worker who is fluent in two languages, performed the back-translation into English to improve the comprehensibility and clarity of the contents of the translated version without seeing the original English version; (4) the researcher and the translator compared the back-translated tool with the original English tool. The final translation was completed by confirming that each item had no difference in meaning. In this process, item no. 4, “If work is less difficult, I am not involved in conversations with family” was revised to “If I have a lot of work, I participate less in conversations with my family.” Other items were used as they were because there were no changes in meaning.

#### 2.4.2. Content Validity

In this study, the validity of the content of the WFBRC-S-K, which had undergone translation and back-translation, was verified by three nursing professors as well as by two men and two women with more than 10 years of work experience. To measure the fitness of the content for each item, the content validity index (CVI) was calculated by obtaining the ratio of the number of experts who selected three or four points to the total number of experts for a specific item in the 4-point scale suggested by Lynn [30] (1 point: irrelevant, 2 points: the relevance cannot be judged without revising the item, 3 points: relevant but the item needs to be revised, and 4 points: very relevant). All 30 items had a CVI of 0.80 or more and were selected without any deleted items.

#### 2.4.3. Preliminary Survey

To clarify the contents of the WFBRC-S-K, a preliminary survey was conducted of 24 office workers, which is 10% of the sampled population. The survey confirmed that no item in the questionnaire was difficult to answer or was not understood. Thus, it was decided that the translated questionnaire would be used.

### 2.5. Statistical Analysis

To analyze the collected data, the IBM SPSS 22.0 (IBM, Armonk, NY, USA) [31] and AMOS 22.0 statistical programs (IBM, Armonk, NY, USA) were used. The general characteristics of the study participants were analyzed using descriptive statistics, and Korean version WFBRC-S’s content validity was confirmed through the analysis of content validity index (CVI). The construct validity was verified for the items in each subcategory through the confirmatory factor analysis (CFA) method provided by the structural equation model. To verify the construct validity of the Korean version of the WFBRC-S, the χ^2^, CMIN/df, root mean square error of approximation (RMSEA), root mean square residual (RMR), standardized root mean square residual (SRMR), goodness of fit index (GFI), incremental fit index (IFI), Tucker–Lewis index (TLI), and comparative fit index (CFI) were obtained. In addition, to verify the convergent validity of the Korean version of the WFBRC-S, the standardized factor loading, critical ratio (C.R.), construct reliability (CR), and average variance extracted (AVE) were used. To verify the discriminant validity, the correlation coefficient and AVE were used. To verify the criterion-related validity, the correlation with the WLBOC-S was verified using Pearson’s correlation. Finally, to verify the reliability of the Korean version of the WFBRC-S, its internal consistency was confirmed using Cronbach’s α value.

## 3. Results

### 3.1. General Characteristics of the Study Participants and Level of Variables

As to gender, 37.0% were male and 63.0% were female. As to age, 55.7% were in their 40s and 26.0% were in their 30s, and 71.0% were 40 years old or older. In terms of education, the majority (37.0%) were college graduates, followed by completers of graduate school courses or higher-level courses, two-year college graduates, and high school graduates, in that order. As for occupation, the majority (39.6%) held managerial positions, followed by white-collar, other, professional, production, and service positions, in that order. As for employment type, the majority (80.9%) were regular workers, followed by non-regular workers (12.8%). As for monthly income, the majority (26.8%) earned KRW 2–3 million. The average working hours of the participants were 39.37 (SD = 12.70) hours, and the average personal call time was 5.86 (SD = 9.31) min (Table 1).

As for the level of the study variables, that of the work–family behavioral role conflict was 89.63 (SD = 14.74), and of the sub-dimensions, 48.99 (SD = 8.27) and 40.82 (SD = 8.05) for work interference with family (WIF) and for family interference with work (FIW), respectively. The absolute value of the skewness of the variables was 3, and the absolute value of the kurtosis was less than 10, which indicate that the normality assumption was satisfied [20].

### 3.2. Validity and Reliability of the Tool

#### 3.2.1. Content Validity

The content validity index verified by a total of seven persons, three nursing professors, and two men and two women with 10 years or more of work experience was 1.0 for all the items, which indicates that the content of all the translated items was valid. Thus, all the 30 items were used in the final questionnaire.

#### 3.2.2. Construct Validity

**Figure 1:**
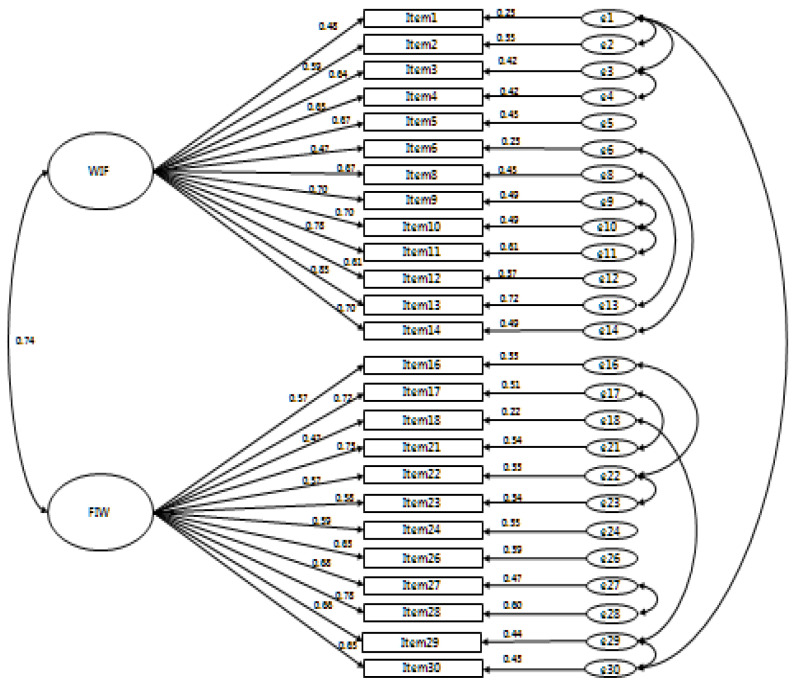
Confirmatory factor analysis.

The confirmatory factor analysis (CFA) was conducted for two sub-areas of the original tool to confirm the construct validity of the WFBRC-S-K. The examination of the standardized factor loading for all the 30 items showed that five items, namely, items 7, 15, 19, 20, and 25, had a standardized factor loading of less than 0.50. The items that lowered the overall model fit were deleted. Then, the standardized factor loading of the remaining 25 items was determined as 0.49–0.81. Among these items, the standardized factor loading of items 6 and 18 were 0.49 and 0.49, respectively, which were below the minimum standard. However, these items were not deleted and were still used for the final analysis (Table 2 and Figure 1) because deleting them might change the meaning of the constituent concept. The examination of the relationships of the items by factor to verify the reliability and validity of the WFBRC-S-K showed that the C.R. of the non-standardized value was in the range of 6.99–9.09, which satisfied the analysis condition of 1.96 or higher.

The examination of the model fit of the WFBRC-S-K, which consists of 25 items (Model 2), showed that the χ^2^ value was 938.29 (df = 274, *p* < 0.001), which indicates that the *p* value was less than 0.05. Since the χ^2^ value increases as the sample size increases, a correct χ^2^ value can be obtained only when the sample size is appropriate. Therefore, the confirmation of the model fit by checking Nomed χ^2^ (CIMIN/df), which is the value obtained by dividing χ^2^ by df (3.42 in this study), showed that Nomed χ^2^ failed to meet the criterion for acceptability of 3 or less. In addition, the model fit indices were RMSEA = 0.10, RMR = 0.05, SRMR = 0.07, GFI = 0.76, IFI = 0.71, TLI = 0.77, and CFI = 0.79, which did not meet the criteria (RMSEA, 0.08–1.00; RMR, 0.05 or less; SRMR, 0.05 or less; GFI, 0.9 or more; IIF, 0.9 or more; TLI, 0.9 or more; CFI, 0.9 or more). Therefore, Model 3 was constructed, which modified Model 2 to increase the model fit by applying the modification index (MI). The analysis of the fitness of Model 3 showed that Nomed χ^2^ (CIMIN/df) dropped to 2.05, and the fitness indices RMSEA (0.07), RMR (0.04), SRMR (0.06), GFI (0.85), IFI (0.91), TLI (0.90), and CFI (0.91) satisfied the minimum standards for all the items. Therefore, the construct validity of the WFBRC-S-K, which consists of two areas with a total of 25 items, was confirmed (Table 3).

#### 3.2.3. Convergent Validity

The convergent validity of the WFBRC-S-K was verified based on the standardized factor loading, AVE, and construct reliability of the 25 items of the tool. The standardized factor loading of the 25 items was 0.49–0.81, which satisfied the minimum standard of 0.50 or more for all items, excluding items 6 and 18, the standardized factor loading values of which were 0.49 and 0.49, respectively. In some literatures, the standard is set at 0.45, in consideration of the possibility that the meaning of the constituent concept will change [21]. Based on this, it was confirmed that the standardized factor loading in this study satisfied the criteria for all items (Table 2). The AVE was 0.51–0.55, which satisfied the minimum standard of 0.50, and the CR was 0.93–0.94, which satisfied the minimum standard of 0.70. Therefore, since the WFBRC-S-K satisfies the minimum standards for standardized factor loading, AVE, and construct reliability, the convergent validity of the items for measuring the work–family behavioral role conflict of Korean office workers was confirmed (Table 2).

#### 3.2.4. Discriminant Validity

To verify the discriminant validity of the WFBRC-S-K, the AVE value of the two constituent concepts and the squared value of the correlation coefficient between the two constituent concepts were compared, and the discriminant validity was considered satisfied when the AVE value was greater than the square of the correlation coefficient. In this study, the correlation coefficient between work interference with family (WIF) and family interference with work (FIW) was 0.65, and the square was 0.43, which is smaller than 0.55, the AVE value of WIF, and 0.51, the AVE value of FIW, therefore confirming the discriminant validity of the WFBRC-S-K (Table 2 and Table 4).

#### 3.2.5. Criterion-Related Validity

To verify the criterion-related validity of the WFBRC-S-K, the relationship with Park and Sohn [28]’s tool, the validity of which was verified by the WLBOC-S in previous studies, was analyzed. The analysis showed that the two tools had a statistically significant negative correlation (r = −0.25, *p* < 0.001). In addition, the work–life balance organizational culture and the sub-factors of the Korean version of the WFBRC-S, specifically WIF (r = −0.22, *p* < 0.001) and FIW (r = −0.23, *p* < 0.001), showed significant negative (−) correlations, and the two sub-factors (r = 0.65, *p* < 0.001) showed a significant positive (+) correlation, therefore confirming the criterion-related validity of the Korean version of the WFBRC-S (Table 4).

#### 3.2.6. Reliability

The confirmation of the internal consistency of the 25 items of the Korean version of the WFBRC-S showed that their Cronbach’s α was 0.94. Additionally, for the sub-factors, the Cronbach’s α of the 13 items under WIF was 0.91, and for the 12 items under FIW, 0.90.

## 4. Discussion

In the case of Korean double-income couples, the role of fathers in housework and parenting is increasing, and the problem of equally maintaining the work–family and family–work balance is no longer limited to mothers [32]. Thus, this study verified the validity and reliability of WFBRC-S-K, including both male and female double-income adults in Korea. In addition, this study attempted to complete the WFBRC-S-K with 25 items of the WFBRC-S by verifying the validity and reliability of the WFBRC-S-K, and to provide a basis for measuring the reliability and validity of the tool.

While previous studies have focused on the development of tools to evaluate subjective perceptions of work–family and family–work conflicts, WFBRC-S was developed in order to evaluate behavioral problems caused by conflicts. Hammer, Bauer, and Grandey [33] suggested that the conflict between work and family causes emotional and behavioral consequences in relation to family, as well as makes it possible to predict work-related outcomes. Based on this, WFBRC-S, which can evaluate even conflicts of behavioral roles, is expected to be a useful tool for finding work–family conflicts compared to the existing tools.

As a result of this study, WFBRC-S-K deleted five items whose standardization factor load was lower than 0.50 out of 30 items in the original tool. Particularly, in the case of question 18, “I went home early because of a personal appointment”, this question was presumed to have been asked because of the difference in organizational culture between Korea and the West. In Korea, a culture in which workers are not accustomed to leaving the office early for personal work is still prevalent because office workers should give priority to the value of work [34]. This question is considered to have a relatively low impact compared to the other questions. In the future, it is necessary to consider replacing this question with an expression such as “I have adjusted my working hours due to family work.” On the other hand, the questions of the measurement tools should be described without expressing accurate opinions or negatives so that the respondents can understand them well [35]. In this regard, it is thought that the questionnaire item 25 of this study, “When there is stress in family life, I am unable to well manage my image shown in the company”, may degrade the subjects’ understanding thereof because there is no further explanation. In addition, the WFBRC-S tool needs to be modified in the case of other deleted question items, taking into account the critical expressions.

The convergent and discriminant validity of the items were also examined. It was confirmed that 13 WIF and 12 FIW items were appropriate for measuring each factor and were distinguished from the items included in other factors. Then, it can be judged that the construct validity of the 25 items has been secured.

To verify the criterion-related validity of the tool, the relationship with the WLBOC-S was analyzed. Significant correlations with both WIF and FIW were found. It means that the greater the balance between work and home life is achieved, the less the conflict, and that the criterion-related validity of the WFBRC-S-K has been secured. However, since the gold standard tool for comparing the validity of WFBRC-S-K could not be found, this study has a limitation that does not reflect such validity, and studies should be repeated in the future in order to address this problem. On the other hand, if a more important meaning is given to the work performed at work than to the work shared at home, the person may come into conflict by thinking that social achievement is hindered by the family. In addition, in the opposite case, if a person does not take good care of their family due to work in the company, it may result in conflicts and problems [36]. In this respect, WFBRC-S-K is thought to be utilized as a useful tool to measure the contents of the conflicts of behavioral roles of work–family and family–work, and to present specific criteria.

Reliability refers to the consistency of the values measured using a specific tool. Generally, the reliability should be more than 0.70 [37]. For this measurement tool, the Cronbach’s α was 0.91 for WIF, 0.90 for FIW, and 0.94 for the entire tool, which are similar to the Coefficient’s α = 0.90 for WIF and FIW of the original tool. In addition, for the two sub-factors, WIF and FIW, the Cronbach’s α values were higher than the Coefficient’s α values of the WIF and FIW in Carlson et al. [22]’s study on the work–family conflict tool, which were 0.83 and 0.86, respectively. This result means that the WFBRC-S-K is internally consistent, so it can be used to measure conflict between work–family behavioral roles of the double-income couple and the effect of such conflict when performing a nursing intervention program.

## 5. Limitations and Significance

As a result of this study, regarding the employment type of the subjects, 80.9% of the subjects were regular workers compared to non-regular workers, thus indicating that the survey was conducted targeting mainly regular workers. In view of the fact that more Korean women are working at small-sized and medium-sized companies rather than at large-sized companies [38], the generalization of these study results is considered to be somewhat limited. On the other hand, since the WFBRC-S-K evaluated in this study was based on the content and format of the original tool developed for a wide range of people, there may be limitations in evaluating subjects with special job characteristics that are different from the job characteristics of general occupations. In addition, the sub-factors of the WFBRC-S-K were analyzed based on the sub-factors organized by the developer, so it is necessary to check them once more in future research.

However, despite these limitations, this study reflected the reality of Korea in order to verify the reliability and validity of the tool. Furthermore, it is believed to be meaningful that the tool comprehensively reflected conflicts by adding behavioral conflicts to the perception of work–family conflicts. In particular, the greatest significance of this study is that this tool can measure the double role burden [39] of women caused by their growing participation rate in economic activities. In addition, this study can be applied to other studies outside the field of nursing, such as to sociological and psychological studies, and it contributes to the establishment of a foundation for preparing the rationale for maintaining work–life balance by reflecting the current situation that places importance on work–family balance. The findings from this study can be used for society and policymakers.

Based on the results of this study, first, it is necessary to apply the WFBRC-S-K to various subjects and industrial sites to identify the conflict between work and family roles and behaviors, and to explore the factors that cause such conflict, and to attempt the experimental studies for intervention. Second, since such conflict is affected by individual general characteristics and various factors in different situations, replication studies with bigger samples are needed, and the studies to verify the consistent effectiveness of the tool are also needed. Third, the measurement tools used in this study may have systematic errors due to bias caused by cultural differences in Korean society, so methods to reduce such errors or mistakes should be considered. Fourth, since the situation of COVID-19 may have influenced the conflict, studies that reflect this are needed.

## 6. Conclusions

In modern society, due to the increase in the number of double-income families and the extension of work due to the widespread use of the internet and smartphones, various work and family role conflicts have arisen. However, the WFBRC-S has been unable to distinguish between individual subjective behavior and behavioral role conflict, and thus, a supplementary tool has been developed. This study intended to confirm the validity and reliability of the WFBRC-S-K and to evaluate its applicability to the Korean general public.

In this study, the WFBRC-S-K was developed using the translation-back translation process, and its content validity, construct validity, convergent validity, discriminant validity, criterion-related validity, and reliability were analyzed. As a result, five of 30 items were deleted in the development of the WFBRC-S-K. The measurement tool developed in this study confirmed the appropriate reliability of the WFBRC-S-K in the reliability evaluation that confirmed the internal consistency of the tool, and satisfactory validity was confirmed through a correlation analysis between the WFBRC-S-K and the WLB organizational culture scale. In the future, it is expected that research that will apply the WFBRC-S-K to the measurement of work–family conflict in nursing and other industrial sites will be actively conducted.

## Figures and Tables

**Table 1:** General characteristics of the study participants.

Characteristics	n (%)	Mean (SD)	Range
Gender			
Female	148 (63.0)		
Male	87 (37.0)		
Age (year)			
≤29	7 (3.0)		
30–39	61 (26.0)		
40–49	131 (55.7)		
≥50	36 (15.3)		
Education level			
≤High school	26 (11.1)		
College	52 (22.1)		
University	87 (37.0)		
≥Graduate school	70 (29.8)		
Occupation type			
Professional	20 (8.5)		
Management position	93 (39.6)		
Service industry	15 (6.4)		
Office job	57 (24.3)		
Blue-collar job	19 (8.1)		
Other	31 (13.2)		
Forms of employment			
Permanent position	190 (80.9)		
Temporary position	30 (12.8)		
Other	15 (6.4)		
Monthly income			
≤200	30 (12.8)		
201–300	63 (26.8)		
301–400	57 (24.3)		
401–500	40 (17.0)		
≥501	45 (19.1)		
Working hours per week (hour)	-	39.37 (12.70)	6–80
Call duration for one time (minute)	-	5.86 (9.31)	0–60

**Table 2:** Analysis of convergent validity of items.

Factors	Items No.	B	SE	β	C.R.	*p*	AVE	CR	Cronbach’s
Work interference with family (WIF)	1	1.00	-	0.51	-	<0.001 *	0.55	0.94	0.91
	2	1.19	0.17	0.61	6.86				
	3	1.32	0.18	0.68	7.28				
	4	1.44	0.20	0.68	7.31				
	5	1.40	0.19	0.67	7.21				
	6	1.12	0.19	0.49	5.99				
	8	1.25	0.18	0.64	7.07				
	9	1.78	0.24	0.72	7.48				
	10	1.55	0.20	0.75	7.63				
	11	1.79	0.23	0.79	7.86				
	12	1.03	0.15	0.60	6.77				
	13	1.88	0.24	0.81	7.93				
	14	1.52	0.21	0.70	7.40				
Family interference with work (FIW)	16	1.00	-	0.58	-	<0.001 *	0.51	0.93	0.90
	17	1.19	0.14	0.73	8.58				
	18	0.83	0.13	0.49	6.43				
	21	1.23	0.14	0.74	8.66				
	22	0.88	0.12	0.59	7.39				
	23	0.89	0.12	0.59	7.41				
	24	1.05	0.14	0.61	7.58				
	26	0.88	0.12	0.62	7.65				
	27	1.15	0.14	0.72	8.49				
	28	1.28	0.14	0.80	9.09				
	29	1.09	0.13	0.69	8.25				
	30	1.05	0.13	0.67	8.09				

* *p* < 0.05.

**Table 3:** Analysis of construct validity.

	χ^2^(*p*)	df	CIMIN/ df	RMSEA	RMR	SRMR	GFI	IFI	TLI	CFI
Model 1	1485.45 *	404	3.68	0.11	0.07	0.09	0.70	0.71	0.68	0.71
Model 2	938.29 *	274	3.42	0.10	0.05	0.07	0.76	0.79	0.77	0.79
Model 3	534.14 *	260	2.05	0.07	0.04	0.06	0.85	0.91	0.90	0.91

* *p* < 0.05.

**Table 4:** Analysis of criterion-related validity.

Scales	WLBOC-S	WFBRC-S-K	WIF	FIW
				r (*p*)
WLBOC-S	1			
WFBRC-S-K	−0.25 *	1		
WIF	−0.22 *	0.82 *	1	
FIW	−0.23 *	0.79 *	0.65 *	1

* *p* < 0.05.
